# Intravirion DNA Can Access the Space Occupied by the Bacteriophage P22 Ejection Proteins

**DOI:** 10.3390/v13081504

**Published:** 2021-07-30

**Authors:** Justin C. Leavitt, Eddie B. Gilcrease, Brianna M. Woodbury, Carolyn M. Teschke, Sherwood R. Casjens

**Affiliations:** 1 School of Biological Sciences, University of Utah, Salt Lake City, UT 84112, USA; justincleavitt@gmail.com; 2 Division of Microbiology and Immunology, Department of Pathology, University of Utah School of Medicine, Salt Lake City, UT 84112, USA; eddie.gilcrease@path.utah.edu; 3 Department of Molecular and Cell Biology, University of Connecticut, Storrs, CT 06269, USA; brianna.woodbury@uconn.edu; 4 Department of Chemistry, University of Connecticut, Storrs, CT 06269, USA

**Keywords:** bacteriophage P22, ejection proteins, intravirion location

## Abstract

Tailed double-stranded DNA bacteriophages inject some proteins with their dsDNA during infection. Phage P22 injects about 12, 12, and 30 molecules of the proteins encoded by genes *7*, *16* and *20*, respectively. After their ejection from the virion, they assemble into a trans-periplasmic conduit through which the DNA passes to enter the cytoplasm. The location of these proteins in the virion before injection is not well understood, although we recently showed they reside near the portal protein barrel in DNA-filled heads. In this report we show that when these proteins are missing from the virion, a longer than normal DNA molecule is encapsidated by the P22 headful DNA packaging machinery. Thus, the ejection proteins occupy positions within the virion that can be occupied by packaged DNA when they are absent.

## 1. Introduction

The short-tailed dsDNA bacteriophage P22 virion contains three proteins, the products of genes *7*, *16* and *20* (called gp7, gp16 and gp20, respectively) that are required for successful delivery of the DNA from the virion into target *Salmonella* host cells [1,2,3,4,5], but the exact functions of these “ejection proteins” (E-proteins) are not fully understood. All three are quantitatively released from virions during the process of DNA delivery into target cells [2,3,5,6]. After their ejection from the virion these proteins are thought to assemble into a hollow conduit that carries the DNA from the virion through the target cell periplasm into the cytoplasm [7,8,9,10]. This idea is strongly supported by the observations that a gp20 homologue encoded by the *Shigella* P22-like phage Sf6 assembles in vitro into a hollow tube-like structure with an internal diameter of 25 Å and length of 150 Å [11]. A similar tube-like structure that is dependent on functional ejection proteins has been observed protruding through the target cell’s periplasm from the base of the tail of infecting P22 virions [12]. Like phages Sf6 and P22, the short-tailed virions of phages T7 and epsilon15 also form trans-periplasmic channels during injection [13,14,15], suggesting that the construction of such conduits from internal proteins is a general feature of *Podoviridae*. Little is known about the nature of such trans-envelope channels, and we note that the proteins thought to build them in P22, T7 and epsilon15 are not recognizably related to each other at the sequence level.

The location of the E-proteins in the P22 virion is not accurately known. The head of the bacteriophage P22 virion contains its ~43,400 bp dsDNA chromosome and is built from 415 molecules of major capsid protein (gp5, the product of gene *5*) and twelve molecules of portal protein (gp1). Its short tail is composed of twelve molecules of gp4, six molecules of gp10, a trimer gp26, which serves to plug the portal complex channel and prevent premature release of the genome, and six trimeric tailspikes (gp9) [16]. Atomic resolution X-ray structures of four of these proteins (gp1, gp4, gp9 and gp26) have been reported, and a robust trace of the gp5 polypeptide chain has been deduced from cryo-electron microscopic (cryoEM) reconstructions [17,18,19,20,21,22]. These five protein structures all fit very well into the cryoEM reconstruction of the virions, yielding a rather well-understood “virtual atomic” view of most of the virion structure. The structure of the tail protein gp10 has not been determined, but its position in the virion at the base of the tail is well established [16,23]. Interestingly, the electron density in asymmetric cryoEM reconstructions of P22 virions is essentially all accounted for by the presence of the above proteins, and there is no clear electron density that might be due the three E-proteins in these virions [16,24,25]. Our best, but still imperfect, estimates of the number of molecules of gp7, gp16 and gp20 present in each P22 virion are 12, 12 and 30, respectively, which amounts to a total E-protein mass of about 3.0 × 10^6^ Da [26]. They should be easily visualized in the cryoEM reconstruction if the E-proteins were to occupy the same position in every virion.

Bryant & King [27] interpreted the ultraviolet radiation damage to the E-proteins that is mediated by DNA-bound acridine to mean that they are in contact with DNA in the virion. Our more recent bubblegram analysis of P22 virions supports this idea and indicates that the E-proteins appear to lie at least in part near the center of the virion and the long interior shaft of the portal protein complex [26]. The failure to visualize them by cryoEM suggests that they are either very flexible and/or occupy different positions in different virions. In this report we show that the P22 E-proteins occupy locations within the virion that can be occupied by DNA when they are absent.

We dedicate this article to the memory of the late Lindsay W. Black, our friend and colleague who pioneered the study of phage virion proteins that are injected with the DNA into target cells [28,29,30,31].

## 2. Materials and Methods

### 2.1. Phage and Bacteria Strains

P22 UC-0937 was the “wild type” parent of the mutants used in this study. It carries wild type DNA packaging genes and packages DNA in a normal, wild type fashion. Isogenic mutant P22 phages were constructed for this study using the galK recombineering methods described in references [32,33,34]. The pKD46 plasmid [35] was present to promote recombinational replacement and was removed by growth at 41 °C before prophage induction. To generate isogenic P22 strains that carry nonsense mutations in the E-protein genes, 1–2 kbp DNA fragments were PCR amplified from the appropriate region of P22 strains UC-0100, -0008, -0009 (Table 1) that contain *amber* mutations *7^−^am*H1375, *16^−^am*N121 and *20^−^am*N20, respectively [1]. These DNAs were used to replace the *galK* cassettes in UB-2276, UB-2274 and UB-2272 to generate versions of prophage P22 UC-0937 in host strain UB-2235 that carry these *amber* alleles (*Salmonella* strains UB-2387, UB 2350 and UB-2366, respectively). The presence of the relevant amber E-protein gene mutation in each of the above phage genomes was confirmed by DNA sequencing of the modified region by the University of Utah High Throughput Genomics Core Facility.

### 2.2. P22 Virion-Like Particle Purification, DNA Preparation and DNA Electrophoresis

P22 virions and virion-like DNA-containing particles were prepared as follows: Prophages were induced from exponentially growing 2 × 10^8^ cells/mL 37 °C L broth cultures of the above lysogens by addition of 0.5 μg/mL mitomycin C or 1.5 μg/mL carbadox and continued shaking for 3–5 h. Cells were concentrated by pelleting and resuspension in L broth, and lysed by shaking with chloroform. Virions and virion-like particles were purified, after cell debris removal by low speed centrifugation, two successive CsCl step gradient centrifugations [36] and dialysis against 1 mM MgCl_2_, 10 mM TrisCl, pH 7.4 buffer. DNA was extracted from virions with a Norgen Phage DNA Isolation Kit (Norgen Biotek Corp, Thorold, Ontario) and subjected to restriction endonuclease cleavage according to the manufacturer’s (New England Biolabs) recommendation. Agarose gel electrophoresis was carried out as described [37].

## 3. Results

### 3.1. Some P22 E-Protein N-Terminal Fragments Are Assembly Proficient

Most previous studies have used nonsense mutations in the P22 E-protein genes and considered them to be completely nonfunctional. However, Adhikari and Berget [38] found that *amber* mutation H1025 in codon 315 of gene *20* produces a 314 amino acid N-terminal fragment (out of 472 amino acids) that assembles into virions. We determined that *20^−^am*N20 is a CAG to TAG change in codon 79 of gene *20*, so it is more likely to be a true null under non-permissive conditions, although it is not known if this short fragment is incorporated into assembling particles. To test whether gp7 or gp16 N-terminal fragments might also be assembly competent we analyzed the proteins present in virion-like particles made by nonsense mutants in these genes by SDS-polyacrylamide gel electrophoresis. Gene *16 amber* mutants N121 and H1273 were found to both be a CAG to TAG change in codon 172 of gene *16,* but no additional band was observed in SDS gels of the particles they produced under non-permissive conditions. On the other hand, we found that DNA-containing virion-like particles produced in non-permissive cells by P22 *7^−^am*H1375 (CAG to TAG change in codon 155 [39]) contain a unique protein band that migrates at the ~15 kDa position in SDS-polyacrylamide electrophoresis gels. This band was isolated and its N-terminal amino acid sequence was determined to be Lys-Gly-Gly-Lys-Gly by the method previously described [40], which matches that of mature gp7 after its cleavage by the host OpdA protease [39]. In addition, we found that deletion of the gp7 N-terminal 20 amino acids has little effect on its functionality; it produces about 60% of the wild type burst of infectious virions. Thus, gp20 binds to procapsids through its N-terminal 314 amino acids, and gp7 binds through its amino acids 21–154. It is not known if either of these fragments has partial function. Because incorporated N-terminal *amber* fragments could affect interpretation of the results described below, we studied E-protein mutants that have the whole gene deleted.

### 3.2. P22 E-Proteins Compete with DNA for Internal Virion Space

P22 is a headful packaging phage whose packaging motor fills all the procapsid’s available internal space with DNA [37,41,42]. After a packaging initiation site (*pac*) on replicated concatemeric phage P22 DNA is recognized and cleaved by the terminase, packaging proceeds unidirectionally from that site for 104% of the genome sequence length, where a non-sequence-specific headful cleavage releases the packaged DNA from the unpackaged portion of the concatemer. Subsequent events in a sequential packaging series proceed from the concatemer end created by the previous headful cut [37,41,42]. Genetic changes that alter the length of the genome sequence do not affect the length of the DNA molecule that is packaged [37,42,43]. In addition, when the head is smaller or some of the internal scaffolding protein fails to exit the normal sized procapsid during DNA packaging, the virion chromosome is shortened [44,45]. Thus, packaged DNA is cut from the replicated concatemer only when the available space within head is full of DNA. If the three E-proteins reside in the interior of the P22 virion, they might occupy space that could be occupied by DNA, and if they were absent, mutant virions missing these proteins might therefore contain longer DNA molecules than wild type virions. This would depend on the intravirion DNA being flexible and not locked into a specific immobile “virion conformation”. Current evidence suggests that tailed phage DNA has substantial mobility within the virion, and that there is no single intravirion conformation that the DNA occupies in all virions ([46,47,48] and references therein).

**Figure 1:**
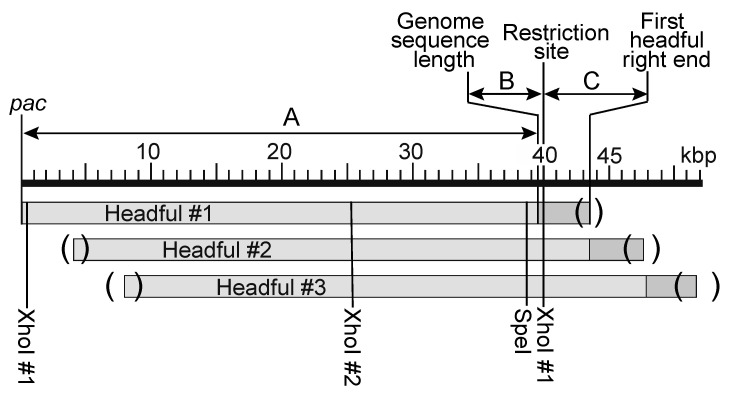
Location of the right end of first DNA headful in P22 packaging series. The first three packaged DNA molecules of a sequential headful series are shown as bars below aligned to the P22 UC-0937 genome kbp scale (its genome sequence is 39,521 bp long); in each headful light gray denotes the leftmost one genome sequence long region and the dark gray denotes the terminal redundancy. Parentheses indicate that there is a range of cleavage sites in different virions due to imprecision in the phage’s headful measuring device. Locations of relevant restriction endonuclease cleavage sites are shown below. The arrows labeled A, B and C above show that the first headful DNA length is determined by the sum of the genome length (arrow A) + distance from there to the restriction site whose cleavage created that fragment (arrow B; XhoI in the case shown) + the first headful right-end restriction fragment length (arrow C).

We therefore examined virion DNA length in P22 mutants missing one or all three of the E-proteins. Pulsed-field gel electrophoresis can be used to determine the size of DNA molecules packaged in the P22 virion DNA, but the accuracy of this method with DNAs this large was insufficient for the purpose of this study. A more accurate method for determination of packaged P22 virion DNA length uses measurement of the length of the right-end restriction fragment from the first headful in a packaging series. P22 right-end fragments give rather diffuse gel bands because the P22 headful measuring device has innate imprecision and packages molecules that are 43,400 ± 750 bp long, where the uncertainty is the actual variation in length, not measurement imprecision [37]. The shortest diffuse right-end restriction fragment DNA band in an agarose electrophoresis gel results from the first headful cleavage in a series, and larger even more diffuse bands are the right-end fragments from subsequent headfuls in packaging series [37,49] (and see Figure 1). The length of the first headful DNA molecule in a packaging series can be simply calculated from the known genome sequence length (arrow A at the top of Figure 1) plus the length of the terminal redundancy. The latter is determined accurately as the sum of arrows B and C in Figure 1, where B is the distance from the right end of arrow A to the restriction site whose cleavage gave rise to the observed right-end fragment (for example, a positive value for XhoI or a negative value for SpeI, see below) and C is the measured length of the first headful’s right-end fragment band.

**Figure 2:**
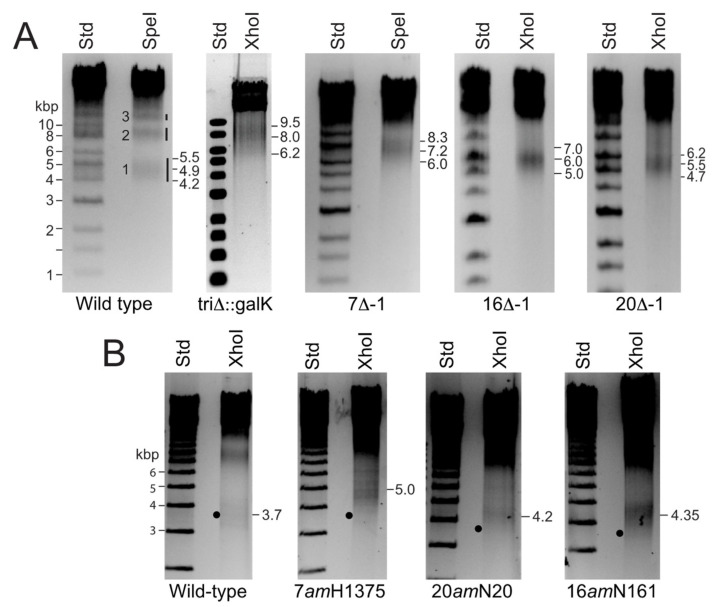
P22 virions without E-proteins contain longer DNA molecules. (Panel **A**) Virion DNA from “wild-type” P22 strain UC-0937 and its derivatives P22 *7^−^*∆-1, P22 *16^−^*∆-1, P22 *20^−^*∆-1 and P22 tri∆::*galK* particles were cleaved with restriction enzyme XhoI or SpeI as indicated and the products displayed in a 0.6% agarose electrophoresis gel; restriction enzymes were chosen that best position the right-end fragment for measurements in the gel. A lane with molecular weight standard DNA fragments (Std) is shown on the left in each gel. To the right of the wild type phage DNA lane, black vertical bars mark the “fuzzy” right end fragments, and their packaging series headful numbers are indicated on the left. The sizes in kbp of the approximate upper and lower boundaries and average size of the first headful right-end bands are indicated at the right of each gel. Note that the genome sequence lengths of the different deletion phages are not the same, so their right end fragment lengths per se do not accurately reflect the amount of DNA packaged (see text). (Panel **B**) DNAs from the *amber* mutant particles indicated below the lanes (see text) were cleaved and the fragments separated as in panel A. Black dots indicate the expected position of the center of first headful right-end band if the packaged DNA were wild-type length.

Restriction endonucleases, SpeI and XhoI, cleave P22 DNA a small number of times (once and twice, respectively) and create first headful right-end fragments 3–8 kbp long that are ideal for such length measurements, since they display the full width of the diffuse right-end fragment band in an uncrowded region of the electrophoresis gel where size measurement is accurate. Wild type P22 UC-0937 and isogenic E-protein defective virion DNAs were cut with SpeI, XhoI or SpeI + XhoI, and the DNA fragments were displayed in agarose electrophoresis gels. Figure 2 shows a representative gel of these mutant DNAs cleaved with SpeI or XhoI. The left portion of panel A of Figure 2 displays the SpeI right-end DNA fragments from headfuls 1, 2 and 3 in the packaging series of P22 UC-237 DNA (the parental “wild type” in these studies; full genotypes of phage and bacterial strains used in this study are listed in Table 1). This P22 strain has been optimized for genetic manipulation using recombineering and shown to assemble fully functional virions and to utilize the normal *pac* site for packaging. This strain’s deletion of the *ImmI-sieA* region increases the length of the terminal redundancy so that right-end fragment bands from the different headfuls in packaging series are well separated in electrophoresis gels see [37]. Measurement of the length of the first headful DNA molecule from P22 UC-237 virions that have all three intact E-proteins present show that it packages 43,400 bp (Table 2) with a headful measurement range (band width) of about ±650 bp; both values are very close to our previously reported P22 wild type headful DNA length measurement [37]. To avoid complications due to the presence of N-terminal *amber* fragments in virions (above), we first examined phages whose ejection protein genes are completely removed by deletion, and Figure 2 panel A displays right-end fragments from the DNAs of P22 phages *7^−^*∆-1, *16^−^*∆-1, *20^−^*∆-1 whose gene *7, 16* or *20* have been deleted, respectively, or P22 tri∆::*galK* where all three ejection protein genes are replaced by a *galK* gene.

The right-end fragments were unequivocally identified by showing that their right end is diffuse (the first headful packaging cut) and their left end is the precise restriction enzyme cut by simple restriction mapping as follows: For example, XhoI cleaves the imprecise ~4900 bp SpeI right-end band of P22 UC-0937 into a sharp ~1050 bp band (sequence predicts 1026 bp if the right end is diffuse and the left end precise) and a diffuse ~3900 bp band that is the same as that produced by XhoI alone (data not shown). The widths of the right-end fragment bands appear to be somewhat larger than wild type in the triΔ::*galK* and *7-am*H1375 (below) mutants. The reason for this is not known. Calculation of first headful DNA lengths for P22 phages *7^−^*∆-1, *16^−^*∆-1, *20^−^*∆-1 and tri∆::*galK* from the sizes of their first headful right-end fragments as described above gave average packaged DNA lengths of 45,100, 44,100, 43,900 and 45,100 which are 1700, 700, 500 and 1700 bp longer than wild-type, respectively (Table 2).

Because these measurements have inherent inaccuracies in estimation of the center of the fuzzy right-end bands and involve calculations from different length genomes (arrow A in Figure 1) due to the different deletion sizes in the different E-protein mutants, we also built the following nonsense (*amber*) mutations into genes *7*, *16* and *20* in the parental P22 UC-0937 by recombineering: *7^−^am*H1375, *16^−^am*N121 and *20^−^am*N20. Their genomes are all the same length, so their right-end fragment lengths directly reflect packaged DNA length. Figure 2 panel B shows DNA electrophoresis gels of these *amber* mutant phage DNAs that have been cleaved by XhoI, and Table 2 lists the increases in packaged DNA length calculated for these mutants. The nonsense mutant particles have increased DNA packaging length values that are very similar to their cognate deletion mutants, in spite of the fact that N-terminal ejection protein fragments can be present in the mutant virions (above).

### 3.3. Does Missing Protein Volume Correlate with Extra DNA Volume?

The above measurements clearly demonstrate that packaged P22 DNA molecules are longer when one or more of the E-protein proteins are absent from the particles, but are the volumes of the extra DNA and the absent proteins in these particles quantitatively related? The internal volume that one bp of DNA occupies in the P22 virion can be estimated empirically as follows: the internal volume of the virion shell is approximated as 96.7 × 10^6^ Å^3^ by a sphere of radius ~285 Å, the average inside radius of the P22 virion head shell [16]. We estimate from the virion structure determined by Tang et al. [16] that the portion of the portal protein dodecamer that is inside the head below this radius to be about 1.6 × 10^6^ Å^3^. Thus, the protein-free volume inside the wild type P22 head is about 92.1 × 10^6^ Å^3^. The DNA that occupies this space is on average 43,400 bp long (above and [37]), so one bp plus its water of hydration and counterions occupies about 2122 Å^3^ inside the P22 virion. Using this value, the calculated volumes occupied by the extra DNA in the particles missing one or all of the E-proteins are listed in Table 3. The total volume of the three E-proteins (above) should be about 3.0 × 10^6^ Å^3^, assuming that one Da of protein occupies 1.2 Å^3^ [50,51].

We previously found that the numbers of the remaining E-proteins that are present in particles when one of their genes is deleted is often somewhat lower than in wild type particles [26]. During this study we used the same method [26] to estimate the volumes of E-proteins in the *7^−^am*H1375, *16^−^am*N121 and *20^−^am*N20 nonsense mutant particles, and these values are listed in Table S1 (in Supplementary Material). The effects of the E-protein *amber* mutations on the amounts of the other two E-proteins in the particles are somewhat less pronounced than for the deletion mutations. Again, assuming that one Da of protein occupies 1.2 Å^3^, the volumes that are theoretically made available by the absence of E-proteins were calculated as the differences between the total wild type E-protein volume, and the volumes calculated for the E-proteins missing from the mutant particles are listed in Table 3. We estimate ≤10% uncertainty in average extra DNA volume (Table 2) and at least that amount of uncertainty in the missing protein volumes, and the ratios of these are thus ±20–30% uncertain. Nonetheless, such uncertainties do not alter any conclusions drawn here.

The calculated volume of the extra DNA in the tri∆::*galK* particles (3.6 ± 0.9 × 10^6^ Å^3^) is very similar to the total E-protein volume (3.0 ± 0.6 × 10^6^ Å^3^), and the values for extra DNA and missing E-proteins are also the same within experimental error for the *16* minus particles. However, the extra DNA and missing protein values do not correspond as well for *7* or *20* mutant particles; the *20* minus particles have a somewhat larger missing protein volume than extra DNA volume, and the *7* minus particles have a larger extra DNA volume than missing protein volume. These apparent inconsistencies could be due to inaccuracies in the measurements of the number of E-proteins per virion (which depend on their specific staining intensities in electrophoresis gels being the same as P22 gp4 and gp1 proteins to which they were compared [26]) or to changes in conformation or location of the remaining E-proteins that affect DNA accessibility when one is not present. Our attempts to use charge detection mass spectrometry [52] to measure the mass of P22 virion-like particles with and without the E-proteins have not led to more accurate values for their numbers per virion.

Previous experimental determinations of tailed phage intravirion DNA hydration have yielded values between 1.07 and 1.50 g H_2_O/g dsDNA [49,53,54]. A value of 0.91 Å^3^/Da for dsDNA (calculated from its partial specific volume of 0.55 cm^3^/g) suggests that an average sized P22 DNA molecule occupies 27.5 × 10^6^ Å^3^. Thus, the 92.1 × 10^6^ Å^3^ value for the total internal volume (above) indicates that internal water occupies about 65 × 10^6^ Å^3^, which in turn gives a value of 1.29 g H_2_O/g dsDNA. This is within the range of previous values, and, given the relatively high precision of our knowledge of P22 virion structure, it is likely the most accurate.

## 4. Discussion

### 4.1. E-Protein Assembly and Intravirion Location

In spite of the extensive research on delivery of dsDNA into target cells by tailed phage virions, this process remains poorly understood in detail, and in phage P22 the assembly and function of the E-proteins still present several poorly understood aspects. First, their assembly into virions is unusual. Unlike nearly all other phage head assembly proteins that have been studied, capsid assembly is not blocked when any of the P22 E-proteins is missing, but proceeds to build apparently normal virions that lack only that protein [1,4,5,55]. Additionally, the ability of at least two of the E-proteins’ N-terminal fragments to assemble into heads is also quite unusual for phage virion proteins [40]. In one other studied case, Black and colleagues showed that T4 E-proteins proteins IpI, IpII and IpIII are also not essential for assembly of DNA-containing virions, and the N-terminal ten amino acids of IpIII are sufficient to program fusion proteins’ assembly into virions [28,56,57].

If any P22 E-protein is missing the other two successfully assemble into procapsids, so each has its own binding site in the assembling particle, but a missing protein can subtly affect the quantity of other E-proteins assembled (above and [26]). The absence of portal protein does not affect E-protein incorporation into procapsids [1,4,5,55]. Coat protein shells made by a scaffolding null mutant do not contain gp7, gp16 or gp20 [4,58], and some point mutations or a short deletion in scaffolding protein block incorporation of gp16 into procapsids [45,59]. Thus, scaffolding protein is necessary for E-protein assembly into procapsids. Scaffold molecules all leave the procapsid when the coat protein shell expands and DNA is packaged [36,55,60], so if E-protein incorporation is mediated by binding to scaffold, it must release them upon its exit. Thus, the location of the E-proteins in procapsids or virions is not predicted from assembly studies, and no obvious E-protein protein density is present in the 3d-reconstructions of either procapsids or virions [16,25]. We note that it was postulated in the original report of the asymmetric reconstruction of the P22 virion that the E-proteins form a tube that extends towards the center of the virion from the portal vertex [61]; however, subsequent crystal structures of the portal protein dodecamer have shown it to have a long C-terminal “barrel” that rather closely matches the size and orientation of the tubular density seen in the virion reconstruction [16]. More recently we used bubblegram technology to show that the virion P22 E-proteins appear to be located somewhere near the center of this portal barrel [26], and the results presented here show that they are inside the virion in space that becomes accessible to DNA when they are absent. Whether the E-proteins reside on the interior or exterior of the portal shaft cannot be deduced from these studies.

### 4.2. A Model for E-Protein Actions

While it is not known how or if the E-proteins are precisely positioned for proper ejection, it is nonetheless clear that they are released from the virion during the DNA injection process [6], that the absence of any of the E-proteins causes failure of DNA to be properly delivered into the cell [1,2,3,4,5], and that without E-proteins the K^+^ release that accompanies DNA injection [62] does not occur (our unpublished results). Recent observations that the E-proteins almost certainly form the periplasm-spanning conduit through which injected DNA passes on its journey to the cell cytoplasm [12] seem to demand that proteins exit the virion before the DNA, and Jin et al. [63] found that when P22 virions are exposed to their purified lipopolysaccharide receptor under certain conditions in vitro the E-proteins are released but the DNA remains in the virion, indicating that E-proteins can be released from the virion before the DNA is released. However, a protein-first release order is somewhat difficult to reconcile with two experimental observations: (i) The gene *26* encoded tail needle serves as the portal channel plug that keeps the DNA in the virion [64,65,66], and when gp26 is not present P22 DNA is packaged normally, but it soon leaks out of the particle (presumably through the open portal channel) even before the infected cells lyse [67,68]. The E-proteins are not released from these particles [1,4,5,55,69], so DNA can leave under these circumstances without E-protein release. (ii) Electron density is present in the portal channel of cryoEM reconstructions of complete P22 virions, and it has been argued that this is due to DNA or E-proteins in the channel [16,24,61]. The narrowness of the portal channel suggests that it should be difficult for even unfolded E-proteins and DNA to pass through the portal channel simultaneously. If DNA occupies the channel in complete virions, it would seem to have to “back up” out of the channel to allow the ejection proteins to pass out first.

**Figure 3:**
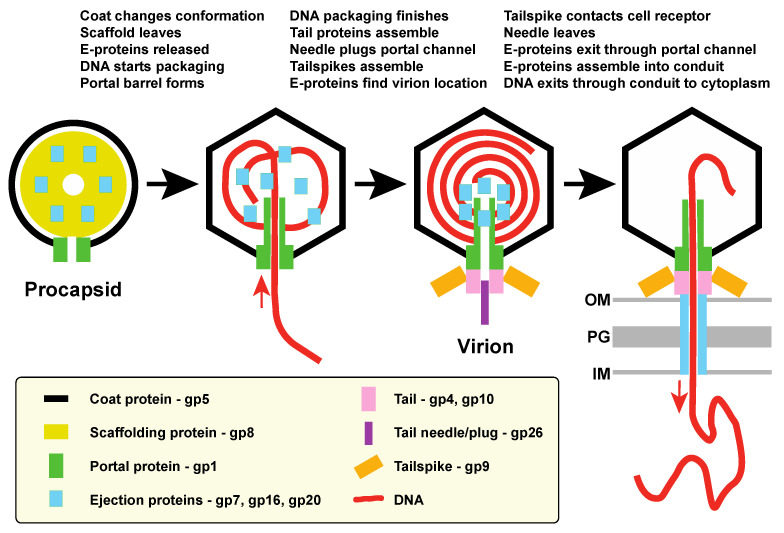
A model for P22 E-protein function. The diagram shows that scaffolding protein recruits the E-proteins into procapsids. The E-proteins are released from scaffold when it exits the structure, and they accumulate near the center of the virion. When the virion binds to a susceptible cell’s receptor, the E-proteins leave the virion through the portal channel and assemble into the trans-periplasmic conduit through which DNA travels into the cell. The order of some steps, for example initiation of DNA packaging, portal barrel formation and E-protein movement to center of particle, have not been resolved (see text). OM, outer membrane; IM, inner membrane; PG, peptidoglycan cell wall.

The above observations may seem contradictory, but the following model for E-protein assembly and function, diagramed in Figure 3, can potentially reconcile them:

1. E-proteins are incorporated into procapsids by interactions with assembling scaffolding protein (and possibly coat protein?), and at least in the cases of gp7 and gp20 it is the N-terminal portion that is responsible for such interactions. Portal protein is not required for E-protein incorporation into virions.
2. During DNA packaging and procapsid maturation, scaffolding protein is released from the particles, coat protein undergoes a major conformational change as the shell expands, and the C-terminal portal barrel appears to form [16,25]. These changes likely release the E-proteins from their initial binding sites and may form new ones.
3. The gp26 trimer needle binds to plug the portal channel and complete the formation of the infectious virion.
4. Upon their release from scaffolding protein, E-proteins move to the near central position in the virion where the bubblegram analysis of virions places them and at least parts of them are in contact with DNA. They could either bind to the newly formed portal barrel or accumulate there because of possibly lower DNA density at the center of the particle. In DNA-filled particles that lack gp26 the E-proteins seem to not yet have reached the final poised-for-ejection location, since DNA can exit but the E-proteins remain associated with the particles. Some consequence of gp26 binding may allow the E-proteins to reach the positions from which they can exit before the DNA during injection. We suggest that this movement may place at least some portion of the E-proteins inside the portal channel. The order of these latter events is unclear, and cryoEM reconstruction of *26* minus particles suggests that the ejection proteins may begin to build the virion proximal portion of the conduit when DNA is spontaneously released under these conditions [69].
  The interior volume of the P22 virion portal vertex channel calculated from the virion structure of Tang et al. [16] (including the inside channel of the portal barrel and the ~120 Å long portion that extends beyond the coat protein shell (see [64])) is only about 0.3 × 10^6^ Å^3^. Thus, at most only about 10% of the total E-protein mass could fit into the channel (making it very unlikely that any E-protein parts actually occupying the channel of the virion would significantly affect the above volume calculations). Nonetheless, it is interesting to note that the 12 gp7 molecules, whose volume is calculated to also be 0.3 × 10^6^ Å^3^, would rather neatly fill the portal vertex channel. It has been suggested that gp7 builds the virion proximal portion of the periplasmic conduit [12], and if so, it seems sensible that it would leave first so as to build the conduit from the virion outward.
5. Upon successful attachment of the virion’s tail to a target cell, the gp26 plug is removed, and E-proteins leave the virion via the portal channel before the DNA is injected and assemble into the trans-periplasm conduit. It is not known if every E-protein molecule in the virion participates in the assembly of the conduit; if E-proteins are present in excess in the virion, it may not be completely debilitating if fewer than the full complement of molecules are present in the virion.
6. DNA passes out of the virion through the cell membranes, peptidoglycan and periplasm into the cytoplasm via this conduit. Curiously, the P22 E-protein conduit appears to be weakly attached to the virion, since it was not present when spent infecting virions were isolated after having released their DNA into cells [6].

Ejection proteins in other tailed phages, including those with long tails, can have functions different from building a conduit such as the one discussed above. These have important functions such as single or multiple subunit RNA polymerases of phage N4 and øKZ [70,71], host RNA polymerase modification by phage T4 ADP-ribosyltransferase Alt protein [72], and phage T4 IpI and phage P1 DarA, DarB and Hdf proteins which interfere with host restriction-modification systems [73,74]. They also have other interesting aspects, for example (i) as many as hundreds of molecules of some E-proteins (T4 phage IpII and IpIII and SPN3US gp53 and gp54—all of unknown function) are injected into target cells [75], (ii) large genome “jumbo” tailed phages may inject as many as a dozen or more different proteins [75,76], and (iii) in some phages putative ejection proteins are very large [77,78]. They include the longest known phage protein, short-tailed phage Pavtok gp*27*, a 5007 amino acid long protein that has partial homology with a P1 E-protein [78]. Their large size makes it seem essential that such proteins pass through the trans-periplasm conduit (and tail in the long-tailed phages) in an at least partially unfolded state. It has been shown that phage T7 ejection proteins gp15 and gp16 unfold at lower than typical temperatures and spontaneously refold in vitro [79,80], and Black and colleagues have shown that foreign proteins fused to T4 ejection protein IpIII are likely unfolded during their injection [28,29]. The study of the mechanism of protein injection by tailed phage virions and the determination of whether it will be technologically advantageous to use such systems to deliver proteins of interest into specific target cells will be fertile ground for future research.

## Figures and Tables

**Table 1:** Bacteria and bacteriophage strains used in this study.

Name	Genotype ^a^	Source
*Salmonella enterica* serovar Typhimurium LT2		
UB-2235	*galK::TetRA*-1,∆Fels-1,∆Fels-2,∆Gifsy-1,∆Gifsy-2 (P22 UC-0937)	b
UB-2272	UB-2235 (P22 UC-0937 with *galK::7*) ^c^	[26]
UB-2274	UB-2235 (P22 UC-0937 with *galK::16*) ^c^	[26]
UB-2276	UB-2235 (P22 UC-0937 with *galK::20*) ^c^	[26]
UB-2278	UB-2235 (P22 UC-0937 with tri∆::*galK*)	[26]
UB-2285	UB-2235 (P22 UC-0937 with *7^−^*∆-1) ^d^	[26]
UB-2288	UB-2235 (P22 UC-0937 with *16^−^*∆-1) ^d^	[26]
UB-2289	UB-2235 (P22 UC-0937 with *20^−^*∆-1) ^d^	[26]
UB-2350	UB-2235 (P22 UC-0937 with *7^−^am*H1375)	this report
UB-2366	UB-2235 (P22 UC-0937 with *16^−^am*N121)	this report
UB-2387	UB-2235 (P22 UC-0937 with *20^−^am*N20)	this report
P22 bacteriophages		
UC-0008	P22 *c1-7, 13^−^am*H101, *2^−^am*N20	[1]
UC-0009	P22 *c1-7, 13^−^am*H101, *16^−^am*N121	[1]
UC-0100	P22 *c1-7, 13^−^am*H101, *7^−^am*H1375	[5]
UC-0937	P22 *c1-7*, *13^−^am*H101, orf25::CamR-EG1, *sieA-*∆1	b

^a.^ UB-2235 in middle column indicates that the strain also carries the *Salmonella* alleles of that strain. b. This *Salmonella* host lacks all functional prophages. P22 UC-0937 contains an *ImmI-sieA* deletion, an *amber* mutation in gene *13,* and the *c1*-7 clear plaque mutation [33,34]; construction details to be published elsewhere; S. Casjens and E. Gilcrease, unpublished). ^c.^ The *galK* cassette neatly replaces the indicated phage gene. ^d.^ These strains have deletions of the entire indicated ejection protein gene.

**Table 2:** Lengths of DNAs packaged by ejection protein mutants.

			Right-End		
	Genome	Restriction	Fragment	Packaged	DNA Length
P22 Phage	Length ^a^	Site Location ^b^	Length ^c^	DNA Length ^d^	Increase ^e^
WT (UC-0937)	39,521	–769 (SpeI)	4650	43,400	–
*20^−^*∆-1	38,161	+257 (XhoI)	5500	43,900	500
*20^−^am*N20	39,521	+257 (XhoI)	4200	44,000	600
*16^−^*∆-1	37,751	+257 (XhoI)	6100	44,100	700
*16^−^am*N121	39,521	+257 (XhoI)	4400	44,200	800
*7^−^*∆-1	38,890	–769(SpeI)	7000	45,100	1700
*7^−^am*H1375	39,521	+257 (XhoI)	5000	44,800	1400
tri∆*::galK*	36,867	+257 (XhoI)	8000	45,100	1700

^a.^ Arrow A (genome) length in Figure 1. The bp lengths of the virion chromosomes of P22 UC-0937 and the four deletion phages were confirmed by Illumina whole genome sequencing. The *amber* mutant phage genomes have the same length as P22 UC-0937. ^b.^ Arrow B length in Figure 1. The restriction enzyme used was chosen to position the right-end fragment in the gel for optimum size measurement. ^c.^ Arrow C (right end fragment) length in Figure 1. Values are the average of three or more determinations of the average length and of the right-end restriction fragments (center of fuzzy gel band). The range of values obtained indicated that the accuracy of this measurement was about ±100 bp when fragments are in the 2–5 kbp range and ± 200–300 bp for larger sizes. ^d.^ Length of DNA packaged is the sum of the lengths of arrows A + B + C in Figure 1 (see text). ^e.^ Increase in length of mutant packaged DNA over the isogenic P22 UC-937 parent that has all three E-proteins in virion.

**Table 3:** Volumes of extra DNA and missing protein in E-protein mutant virions.

	Average	Volume ^b^	Volume ^c^	
	bp Length	of “Extra”	of “Missing” E-	
P22 Phage	Increase ^a^	DNA (Å^3^ × 10^−6^)	Proteins (Å^3^ × 10^−6^)	Volume Ratio ^d^
*20^−^*∆-1	500	1.1	2.1	0.52
*20^−^am*N20	600	1.3	2.4 (2.1) ^e^	0.54 (0.62) ^e^
*16^−^*∆-1	700	1.5	1.5	1.0
*16^−^am*N121	800	1.6	1.3	0.8
*7^−^*∆-1	1700	3.6	2.3	1.6
*7^−^am*H1375	1400	2.9	1.5 (1.8) ^e^	2.0 (1.6) ^e^
tri∆::*galK*	1700	3.6	3.0	1.2

^a.^ Increased length over wild type DNA. Values are from Table 1. ^b.^ Calculated as described in the text. ^c.^ For each mutant particle, the average total E-protein volume was calculated from the numbers of gp7, gp16 and gp20 molecules present in at least three determinations using value of 1.2 Å^3^/Da for protein (see text). Note that incorporation of each E-protein into mutant particles is partially dependent on the other E-proteins present (see text). ^d.^ “Extra DNA”/”Missing E-protein” volume ratio. ^e.^ Values in parentheses assume that that *amber* fragments are present in mutant the particles in the same number as full-length proteins in wild type virions.

## Data Availability

Data from this study was not submitted to public databases.

## Supplementary Materials

The following are available online at https://www.mdpi.com/article/10.3390/v13081504/s1, Table S1: Quantitation of E-proteins in *amber* mutant particles by SDS-PAGE.
