# Supplementary material for: Intravirion DNA Can Access the Space Occupied by the Bacteriophage P22 Ejection Proteins
Source: Viruses. 2021 Jul 30;13(8):1504. doi: 10.3390/v13081504 (PMC8402733; doi:10.3390/v13081504)
Supplement: Supplementary file 1 [file viruses-13-01504-s001.zip › viruses-1302563-supplementary.pdf]

SUPPLEMENTARY MATERIAL

Table S1. Quantitation of E-proteins in *amber* mutant particles by SDS-PAGE

Phage	Strain name	gp20	gp16	gp7
Wild type	UC-0937	1.0	1.0	1.0
20^{-}◎-1	UC-2289	0	0.5±0.1	0.83±0.1
20^{-}*am*N20	UC-2387	0	0.4±0.2	1.0±0.2
16^{-}◎-1	UC-2288	0.75±0.1	0	0.75±0.1
16^{-}*am*N121	UC-2366	1.00±0.1	0	1.0±0.1
7^{-}◎-1	UC-2285	0.17±0.2	0.5±0.2	0
7^{-}*am*H1375	UC-2350	0.5±0.1	0.7±0.1	0

Values in table are the fraction of wild type numbers of molecules/virion determined as in Wu *et al.* (2016) mBio 7, e01152-16. The values for the deletion (◎) phages are from that report. Uncertainties indicate range of values from three or more determinations.
